# Maternal and offspring intelligence in relation to BMI across childhood and adolescence

**DOI:** 10.1038/s41366-018-0009-1

**Published:** 2018-01-30

**Authors:** Christina Wraw, Ian J. Deary, Geoff Der, Catharine R. Gale

**Affiliations:** 1 Centre for Cognitive Ageing and Cognitive Epidemiology, Department of Psychology, University of Edinburgh, 7 George Square, Edinburgh, SCT EH8 9JZ UK; 2 MRC/CSO Social & Public Health Sciences Unit, University of Glasgow, 200 Renfield Street, Glasgow, G2 3QB UK; 3 MRC Lifecourse Epidemiology Unit, University of Southampton, Southampton General Hospital, Southampton, SO16 6YD UK

## Abstract

**Objective:**

The present study tested the association between both mothers’ and offspring’s intelligence and offspring’s body mass index (BMI) in youth.

**Method:**

Participants were members of the National Longitudinal Survey of Youth 1979 (NLSY-79) Children and Young Adults cohort (*n* = 11,512) and their biological mothers who were members of the NLSY-79 (*n* = 4932). Offspring’s IQ was measured with the Peabody Individual Achievement Test (PIAT). Mothers’ IQ was measured with the Armed Forces Qualification Test (AFQT). A series of regression analyses tested the association between IQ and offspring’s BMI by age group, while adjusting for pre-pregnancy BMI and family SES. The analyses were stratified by sex and ethnicity (non-Black and non-Hispanic, Black, and Hispanic).

**Results:**

The following associations were observed in the fully adjusted analyses. For the non-Blacks and non-Hispanics, a SD increment in mothers’ IQ was negatively associated with daughters’ BMI across all age-groups, ranging from *β* = −0.12 (95% CI −0.22 to −0.02, *p* = 0.021) in late childhood, to *β* = −0.17 (95% C.I. −0.27 to −0.07, *p* = 0001), in early adolescence and a SD increment in boys’ IQ was positively associated with their BMI in early adolescence β = 0.09 (95% CI 0.01–0.18, *p* = 0.031). For Blacks, there was a non-linear relationship between mothers’ IQ and daughters’ BMI across childhood and between girls’ IQ and BMI across adolescence. There was a positive association between mothers’ IQ and sons’ BMI in early adolescence (*β* = 0.17, 95% CI 0.02–0.32, *p* = 0.030). For Hispanic boys, there was a positive IQ-BMI association in late childhood (*β* = 0.19, 95% CI 0.05–0.33, *p* = 0.008) and early adolescence (*β* = 0.17, 95% CI 0.04–0.31, *p* = 0.014).

**Conclusion:**

Mothers’ IQ and offspring’s IQ were associated with offspring’s BMI. The relationships varied in direction and strength across ethnicity, age group and sex. Obesity interventions may benefit from acknowledging the heterogeneous influence that intelligence has on childhood BMI.

## Introduction

Rates of childhood obesity have increased over recent decades. Between 1980 and 2012, in the United States, the percentage of obese children aged 6–11 years increased from 7% to ~18%, and the percentage of obese teenagers aged 12–19 years increased from 5% to ~21% [1, 2]. Being obese in childhood is associated with childhood comorbidities [3–5] and with an increased risk of being overweight in adulthood [6]. It is a matter of public health importance to identify the factors that impact the risk of having a high body mass index (BMI) in early life. The risk factor of interest in the present study is both mothers’ and offspring’s intelligence.

Findings from many studies suggest that there is a robust relationship between lower intelligence in youth and higher BMI, obesity, and weight gain in adulthood [7–11].

More recently, research has focused on analyzing the association between childhood intelligence and early life BMI. Findings from some studies suggest that lower intelligence in early life is associated with higher BMI and a higher risk of obesity in childhood and adolescence [12–14]. Goldberg [12] found that lower intelligence was associated with increased odds of being obese for both boys (odds ratio (OR) = 1.44, 95% confidence interval (CI) 1.36–1.52) and girls (OR = 1.61, 95% CI 1.51–1.73), using data from the 404,922 adolescents who responded to the Israeli military draft board. Other studies have not found a significant association between general intelligence and BMI in childhood [14–16].

The studies cited above are limited because most analyses were based on a single measure of both childhood intelligence and BMI that was taken at a similar point in time and can only indicate the nature of the IQ-BMI relationship at a single year of age [5, 12–15, 17, 18], which likely gives an incomplete picture of the relationship.

One of the aims of the present study was to analyze the association between intelligence and BMI in four, overlapping, cross-sectional samples at different stages of childhood and adolescence.

In order to establish whether or not intelligence is a risk factor for a high BMI in childhood the possibility of reverse causality must be addressed. Findings from two studies suggest that a high BMI is not a risk factor for low cognitive ability [19, 20]. Afzal and Gortmaker [20] tested the effect of childhood BMI on childhood cognitive ability. They used two cohorts (cohort 1, *n* = 2672 and cohort 2, *n* = 1991) from the National Longitudinal Survey of Youth 1979–Child and Young Adults cohort. BMI was the predictor variable. They did not find any significant results after adjusting for time invariant traits on cognitive test scores [20]. One weakness of this study is that the analyses were based on a fixed-effect model, which does not account for the possibility of cognitive ability impacting the risk of being overweight or obese. This study was further limited because it examined the implications of being either obese or not obese on cognitive performance rather than testing this relationship across the full range of BMI.

Factors that influence childhood BMI may span generations. Findings from previous studies suggest that higher parental IQ is associated with a lower risk of poor health outcomes in offspring [21, 22]. However, only one known study has looked at the association between parental intelligence and offspring BMI. Whitley et al. [22]., analyzed the relationship between parental IQ, measured at age 11, and BMI in children aged 4–18 years in 2268 parent-child pairs but did not find any significant associations. Notwithstanding these null findings, there are reasons to believe that parental intelligence might be associated with offspring’s BMI. Intelligence in youth is associated with adult BMI [7–11]. Pre-pregnancy BMI has been found to be associated with offspring’s weight in infancy and BMI in childhood [23–25]. Therefore, maternal BMI may lie on a pathway that links maternal intelligence with offspring’s BMI.

The second aim of the present study was to analyze the relationship between mothers’ intelligence in youth and offspring’s BMI at different stages of childhood and adolescence.

## Methods

### Sample

#### Mothers

The data were derived from two related cohorts. One of the cohorts was the National Longitudinal Survey of Youth 1979 (NLSY-79). The initial sample of the NLSY-79 was representative of non-institutionalized young people who lived in the United States. It was a random household sample and consisted of 12,686 individuals aged 14–21 years on 31st of December 1978. The respondent gave verbal consent to participate at the beginning of the interview [26]. The NLSY-79 study has been described in detail elsewhere [27]. A total of 4932 women from this survey had children and were followed up in the NLSY-79 Children and Young Adults survey [28]. The institutional review boards of Ohio State University and the National Opinion Research Center at the University of Chicago granted ethics approval for the NLSY.

#### Children

Data were also derived from the NLSY-79 Children and Young Adults survey, which consists of the biological offspring of the mothers of the NLSY-79 cohort. The initial interviews were held in 1986 and have been conducted biennially since then. The children in the study were born between 1973 and 2007. In total, 11,512 children and young adults have completed the survey. Parents gave permission for their child to be interviewed [26].

#### Ethnicity

The nomenclature used in the NLSY-79 documentation to refer to ethnic group (non-Black and non-Hispanic, Black, and Hispanic) has been adopted in the current study. Offspring’s ethnicity was based on the ethnicity reported by the mothers in the NLSY-79 [28].

### Measures

#### Age groups

The following age groups were used in the present analyses:

- Middle childhood: ages 5–7 years
- Late childhood: ages 8–10 years
- Early adolescence: ages 11–13 years
- Middle adolescence: ages 14–18 years

Age group cutoff points were based around stages of physical development [6, 29, 30]. Stages of cognitive development were not considered because the PIAT scores were age-standardized [28]. Each child was only included once per age group. In cases where a child was surveyed more than once in a given age range the latest wave was used. Each age group included data on a different set of children, but many children had information in more than one age group.

#### BMI

Weight in kilograms was divided by height in meters squared to make the BMI variables.

##### Mother’s pre-pregnancy BMI

Maternal BMI was based on pre-pregnancy weight in the present study because it is a good indicator of women’s typical BMI and their BMI throughout pregnancy [31]. This measure was based on self-reports of weight, immediately before each pregnancy, and height. The measure of height from 1985 was selected because the respondents would have reached their adult height [32]. Self-reports of height and weight tend to be accurate reports of actual height and weight [33].

##### Offspring’s BMI

Offspring’s height and weight measurements were either measured at the interview or were recalled by the mother (Table S1). To overcome the difficulty of using raw childhood anthropometric data to compare the BMIs in youth, BMI was age and sex standardized using the *zanthro* transformation method of Cole et al. [34]. The WHO 2007 growth reference charts were used as the reference population for this transformation [34].

#### Intelligence

##### Mother’s Intelligence

The mother’s measure of intelligence was the Armed Forces Qualification Test (AFQT), 1989 re-normed version. Mothers were aged 15–23 years when they sat this test in 1980. The AFQT is composed of four subtests: Arithmetic reasoning, mathematics knowledge, word knowledge and paragraph comprehension. The test had a total of 105 items and a time limit of 84 min [35]. AFQT is predictive of academic and job performance [36]. The AFQT variable used in the present study was downloaded from The Bell Curve Page [37]. This measure was scored as a percentile, and was then *z*-scored.

##### Children’s Intelligence

Offspring’s measure of intelligence was the Peabody Individual Achievement Test (PIAT). It was administered to children aged 5–14 years. The present study included the three following PIAT sub-scales: reading comprehension, reading recognition, and mathematical ability [28, 38]. The reading recognition sub-scale consists of 84 multiple-choice questions and measures the ability to pronounce and recognize words. The mathematics sub-scale consists of 84 multiple-choice questions and measures the child’s level of mathematical ability. The reading comprehension sub-scale consists of 66 items and measures children’s ability to obtain meaning from sentences that they read silently [28, 38]. The exams are not timed [28, 38]. The PIAT has high validity and reliability [28, 38]. All three scales were age standardized and each normed sub-scale had a mean score of 100 and a standard deviation of 15.

For late childhood and early adolescence, the three sub-scales (mathematics, reading comprehension, and reading recognition) were averaged and a composite PIAT variable was created. For middle childhood, the composite measure was made from the mathematics and reading recognition sub-scale because the reading comprehension sub-scale had a high proportion of missing values (25%) at this age. Some children were not eligible to complete the reading comprehension test because they scored below 19 on the reading recognition sub-scale. The two-test PIAT composite measure correlated with the three-test PIAT composite measure with *r* = 0.96 for the boys and *r* = 0.97 for the girls in middle childhood.

#### Socioeconomic status

The composite family socioeconomic (SES) index was the average of *z*-scored educational attainment and income. Education was a measure of mothers’ highest grade completed, for each survey year. Data was not available for fathers’ education. Income was a measure of total net household income from all sources from both the mother and her partner, for each survey year. A separate SES variable was created for each age group.

## Analyses

A series of linear regression analysis were run across middle childhood, late childhood, early adolescence and middle adolescence to answer the following questions.

1a. Is childhood IQ associated with BMI, upon adjusting for the child’s age?

1b. Is this association significant upon further adjustment for mother’s IQ, mother’s pre-pregnancy BMI, and family SES?

2a. Is maternal IQ associated with offspring’s BMI, upon adjusting for the offspring’s age?

2b. Is this association significant upon further adjustment for offspring’s own IQ, their mother’s pre-pregnancy BMI, and family SES?

3. Do these associations vary by sex and ethnic group?

We analyzed the sexes separately because previous studies have found the IQ-BMI association to be stronger in females than in males [7, 12]. Ethnicity is a known covariate of childhood BMI [39–41] so we also analyzed the ethnic groups separately. The non-Blacks and non-Hispanics made up the largest ethnic group so they were used as the reference group. All measures were normally distributed.

To test whether or not early life environmental factors, other than maternal pre-pregnancy BMI, confounded or mediated the offspring/maternal IQ-BMI association, we ran an additional set of analyses that included adjustment for birth weight, gestation length, and infant feeding [42–44].

Family SES was adjusted for because previous studies have found indicators of family SES (i.e., parental education, occupation, and/or income) to be negatively associated with childhood BMI [45–47]. To account for inflation, we also adjusted for the year that income was recorded. Goldberg [12] found evidence of an interaction effect between childhood intelligence and SES on childhood BMI such that the associations between lower intelligence and an increased risk of being obese were stronger for adolescents from a high SES (OR 1.61) than a low SES (OR 1.28); therefore, we tested for an interaction to see if the relationship between childhood intelligence and offspring BMI varied by family SES.

Offspring’s intelligence and BMI were taken from the same age group for all of the offspring IQ-BMI analyses except for the analyses for middle adolescence (age 14–18 years) BMI, which included a measure of childhood intelligence from early adolescence (age 11–13 years). Although children sat the PIAT across the ages of 5–14 years, by the age of 14 years the number of tests that were completed dropped and ~50% of values were missing. No PIAT scores that were recorded in middle adolescence were included in the analyses. To test for a nonlinear association between IQ and BMI, mothers’ squared AFQT score and offspring’s squared PIAT score were included in the models.

Additional sets of multinomial logistic regression analyses tested the above associations between intelligence and BMI category, across age group, ethnicity and sex.

### Code availability

The STATA-13 code used in the present study is available upon request.

## Results

The original sample consisted of 4932 mothers and 11,512 offspring, 51% were boys (*n* = 5876). The different sets of analytical samples for each age group were based on the mothers and their offspring who had complete data for the mothers’ IQ, family SES, pre-pregnancy BMI, and offspring’s age, IQ, and BMI, separately, for each age and ethnic group (Tables 1 & 2). BMI varied significantly by ethnic group. Non-Black and non-Hispanic children tended to have the lowest BMI (Table 2) and the lowest percentage of overweight and obese youth (Table S2).

**Table 1:** Descriptive statistics for sample size, AFQT, BMI pre-pregnancy, net family income, maternal education and offspring’s age groups

	Obs	Mean	SD
Total number of mothers^a^	4932		
Total number of children^b^	11,512		
AFQT	4680	−0.35	0.99
Mothers pre-pregnancy BMI kg/m^2 c^	9400	22.99	4.67
Total net family income^c^			
When child was of age 5–7 years	7275	$45,875	$79,241
When child was of age 8–10 years	7281	$48,378	$71,180
When child was of age 11–13 years	6818	$53,362	$71,509
When child was of age 14–18 years	7003	$60,230	$70,839
Mother’s total years of education^c^			
When child was of age 5–7 years	8296	12.63	2.45
When child was of age 8–10 years	8295	12.63	2.48
When child was of age 11–13 years	8096	12.72	2.52
When child wasof age 14–18 years	7278	12.85	2.49
Child’s sex			
Male	5876		
Female	5635		
Age groups^d^			
Girls			
Middle childhood (5–7 years)	4059	6.86	0.67
Late childhood (8–10 years)	4110	9.87	0.67
Early adolescence (11–13 years)	3894	12.83	0.68
Middle adolescence (14–18 years)	3810	17.06	1.03
Boys			
Middle childhood (5–7 years)	4252	6.86	0.67
Late childhood (8–10 years)	4216	9.86	0.69
Early adolescence (11–13 years)	3973	12.90	0.68
Middle adolescence (14–18 years)	3966	17.05	1.04

^a^ The total number of mothers represents the total number of mothers that participated in the Children and Young Adults survey between the years 1986 and 2012

^b^ The total number of children represents all of the children that responded to the Children and Young adult survey between the years 1986 and 2012

^c^ Mothers pre-pregnancy BMI, education, and income for each age group have high number of observations because these scores were recorded separately for each pregnancy/child

^d^ Each child was counted once per age group. Many children were included in more than one age group

**Table 2:** Descriptive statistics of ethnicity for boys and girls by age group and standardized BMI scores for boys and girls by age group and ethnicity

	Non-Black and non-Hispanic	Black	Hispanic		All Ethnic Groups
Observations across ethnicity, age group, and sex	Obs (%)				
Girls					
Middle childhood	2032 (50)	1190 (29)	835 (21)		
Late childhood	1992 (49)	1286 (31)	830 (20)		
Early adolescence	1799 (46)	1276 (33)	818 (21)		
Middle adolescence	1718 (45)	1285 (34)	806 (21)		
Boys					
Middle childhood	2166 (51)	1196 (28)	889 (21)		
Late childhood	2031 (48)	1278 (30)	904 (22)		
Early adolescence	1839 (46)	1265 (32)	869 (22)		
Middle adolescence	1804 (45)	1279 (33)	883 (22)		
Mean BMI across ethnicity, age group, and sex					
	BMI^a^ mean (SD) *n*			ANOVA	BMI^a^ mean (SD) *n*
Girls BMI					
Middle childhood	16.20 (2.70) *n* = 1 947	17.08 (3.64) *n* = 1 127	16.67 (3.27) *n* = 782	29.08 (*p* < 0.001)	16.55 (3.14) *n* = 3 856
Late childhood	18.27 (3.78) *n* = 1 944	19.56 (4.92) *n* = 1 233	18.84 (4.31) *n* = 804	34.81 (*p* < 0.001)	18.78 (4.30) *n* = 3 981
Early adolescence	20.73 (4.37) *n* = 1 762	22.79 (5.75) *n* = 1 243	21.73 (4.87) *n* = 806	63.09 (*p* < 0.001)	21.61 (5.04) *n* = 3 811
Middle adolescence	22.90 (4.74) *n* = 1 713	25.25 (5.84) *n* = 1 268	23.72 (4.83) *n* = 793	76.05 (*p* < 0.001)	23.86 (5.25) *n* = 3 774
Boys BMI					
Middle childhood	16.30 (2.74) *n* = 2 085	16.75 (3.22) *n* = 1 157	16.77 (3.40) *n* = 860	11.83 (*p* < 0.001)	16.53 (3.03) *n* = 4 102
Late childhood	18.30 (3.77) *n* = 1 980	18.83 (4.51) *n* = 1 242	19.01 (4.20) *n* = 865	11.43 (*p* < 0.001)	18.61 (4.11) *n* = 4 087
Early adolescence	20.80 (4.50) *n* = 1801	21.39 (5.10) *n* = 1234	21.32 (5.03) *n* = 844	6.71 (*p* = 0.001)	21.10 (4.82) *n* = 3 879
Middle adolescence	23.83 (4.69) *n* = 1 795	24.33 (5.19) *n* = 1 274	24.41 (5.06) *n* = 873	5.67 (*p* = 0.003)	24.12 (4.95) *n* = 3 942

The number of observations are lower in the bottom half of the table than they are in the top half of the table because some children are missing BMI measures at particular ages

^a^ BMI = (kg/m^2^), all scores are raw and un-transformed

The correlations between the offspring BMI and the study covariates are displayed in Supplementary Table 3 (girls) and Supplementary Table 4 (boys). The majority of the covariates were significantly correlated with each other and with offspring BMI.

### Girls’ linear regression results

**Fig. 1:**
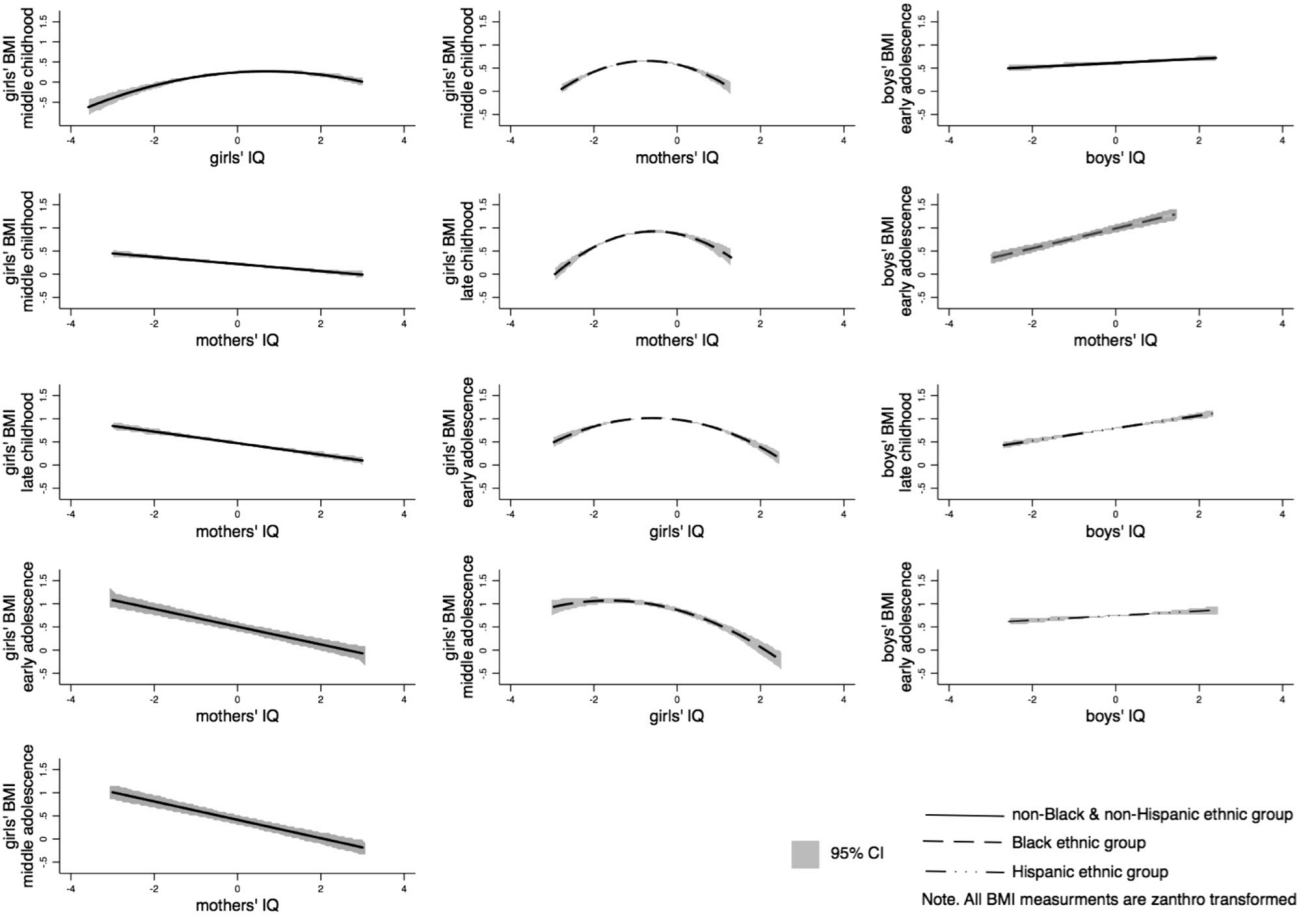
Fitted estimates of the significant associations between both girls’ and boys’ BMI and offspring’s/maternal IQ across age and ethnic groups, adjusted for offspring’s/maternal IQ, offspring’s age at interview, maternal pre-pregnancy BMI, and SES (net family income, year income was recorded, and mothers’ education)

The top of Table 3 displays the results of the linear regression for non-Black and non-Hispanic girls’ BMI on their own IQ and their mothers’ IQ, with and without adjustment for potential confounding and/or mediating factors. In the age-adjusted analyses, mothers’ IQ was negatively associated with daughters’ BMI in both childhood and adolescence (Table 3). The associations ranged from beta = −0.08 in middle childhood to beta = −0.20 in middle adolescence, per standard deviation increment in mothers’ IQ. In the fully adjusted models, all four of the maternal IQ-BMI associations remained significant and there was little change in effect size (Fig. 1). For the result of the age-adjusted analyses of non-Black and non-Hispanic girls IQ-BMI association, a negative linear association was found in middle adolescence (beta = −0.10). There was also a curvilinear association in middle childhood (linear beta 0.07, non-linear beta = −0.05). A standard deviation increment in IQ was associated with a higher BMI up until the average IQ score; beyond this point a standard deviation increment in IQ was associated with a slightly decreasing BMI. In the fully adjusted analyses, the linear association was attenuated to the null but the non-linear association remained significant (Fig. 1). There were no other significant maternal/offspring IQ-BMI associations for non-Black and non-Hispanic girls.

**Table 3:** Regression analyses of the relation between an SD increase in IQ and non-Black/non-Hispanic girls’ and boys’ BMI in childhood and adolescence adjusting for potential confounding and/or mediating variables

	Middle childhood			Late childhood			Early adolescence			Middle adolescence		
	*N*	Beta (95% CI)	*P*-value	*N*	Beta (95% CI)	*P*-value	*N*	Beta (95% CI)	*P-*value	*N*	Beta (95% CI)	*P*-value
**Girls**												
*Girls IQ* ^*a*^												
Baseline model	1458	**0.07 (−0.01 to 0.14)**	**0.067**	1430	−0.03 (−0.11 to 0.05)	0.473	1295	−0.07 (−0.14 to 0.01)	0.084	1193	**−0.10** **(−0.17 to −0.02)**	**0.013**
Quadratic coefficient		**−** ***0.05*** ***(*** **−** ***0.10 to*** **−** ***0.002)***	***0.042***									
Fully adjusted model		**0.08 (0.004 to 0.15)**	**0.040**		0.01 (−0.07 to 0.10)	0.825		0.02 (−0.06 to 0.10)	0.572		−0.02 (−0.10 to 0.06)	0.622
Quadratic coefficient		**−** ***0.05 (*** **−** ***0.10 to*** **−** ***0.01)***	***0.023***									
*Mothers IQ* ^*b*^												
Baseline model		**−0.08 (−0.16 to −0.01)**	**0.037**		**−0.13 (−0.21 to −0.04)**	**0.002**		**−0.19 (−0.27 to −0.11)**	**<0.001**		**−0.20 (−0.28 to −0.12)**	**<0.001**
Fully adjusted model		**−0.14 (−0.24 to −0.05)**	**0.004**		**−0.12 (−0.22 to −0.02)**	**0.021**		**−0.17 (−0.27 to −0.07)**	**0.001**		**−0.16 (−0.26 to −0.06)**	**0.002**
**Boys**												
*Boys IQ* ^*a*^												
Baseline model	1533	−0.02 (−0.09 to 0.06)	0.691	1435	−0.04 (−0.13 to 0.04)	0.287	1325	0.05 (−0.03 to 0.13)	0.230	1192	−0.01 (−0.08 to 0.06)	0.772
Fully adjusted model		0.04 (−0.04 to 0.12)	0.304		0.01 (**−**0.08 to 0.10)	0.790		**0.09 (0.01 to 0.18)**	**0.031**		0.05 (−0.03 to 0.13)	0.229
*Mothers IQ* ^*b*^												
Baseline model		**−0.11 (−0.19 to −0.03)**	**0.008**		−0.05 (−0.13 to 0.04)	0.279		−0.01 (−0.09 to 0.08)	0.877		−0.03 (−0.10 to 0.05)	0.521
Fully adjusted model		−0.09 (−0.19 to −0.02)	0.100		−0.03 (−0.14 to 0.07)	0.535		−0.02 (−0.12 to 0.08)	0.698		0.003 (−0.09 to 0.10)	0.946

Baseline Model: PIAT or AFQT and child age Fully adjusted Model: PIAT, AFQT, child age, mothers’ pre-pregnancy BMI, family SES (net family income, year income was recorded, and maternal education)

^a^ PIAT was the measure of offspring’s intelligence

^b^ AFQT was the measure of mothers’ intelligence

*Note*: Values that are in bold are statistically significant. The quadratic coefficients and *p*-values have been italicised.

Some of the significant results for the Black and Hispanic girls differed from the non-Black and non-Hispanic girls’ results. See Table 4 for a summary of these results and Table S5 for the full results. In the age-adjusted analyses for the Black ethnic group, there was a curvilinear association between mothers’ IQ and daughters’ BMI in middle (linear beta = −0.21, non-linear beta = −0.14) and late (linear beta = −0.18, non-linear beta = −0.16) childhood and a curvilinear association between girls’ own IQ and BMI in early (linear beta = −0.11, non-linear beta = −0.09) and middle adolescence (linear beta = −0.25, non-linear beta = −0.07). All four of these non-linear associations remained significant in the fully adjusted models (Fig. 1). For the girls in the Hispanic ethnic group, a standard deviation increment in girls’ IQ and mothers’ IQ was negatively associated with girls’ BMI in adolescence. Each of these IQ-BMI associations were attenuated to the null in the fully adjusted analyses (Table 4 and Tables S5).

**Table 4:** Summary results from the regression analyses of the relation between an SD increase in IQ and Black and Hispanic girls’ and boys’ BMI in childhood and adolescence upon full adjustment for PIAT, AFQT, child age, mothers’ pre-pregnancy BMI, family SES (net family income, year income was recorded, & maternal education) analyses

	Middle childhood			Late childhood			Early adolescence			Middle adolescence		
	*N*	Beta	*P*-value	*N*	Beta	*P*-value	*N*	Beta	*P*-value	*N*	Beta	*P*-value
**Girls**												
*Black*												
Girls IQ^a^	819	−0.05	0.393	886	−0.04	0.445	863	−**0.12**	**0.043**	**852**	−**0.22**	**<0.001**
^c^								−***0.08***	***0.023***		−***0.07***	***0.038***
Mothers IQ^b^		**−0.20**	**0.182**		**−0.25**	**0.078**		0.06	0.438		−0.04	0.610
^c^		***−0.17***	***0.011***		***−0.20***	***0.001***						
*Hispanic*												
Girls IQ^a^	507	0.03	0.632	534	0.10	0.143	521	−0.02	0.754	480	−0.07	0.213
Mothers IQ^b^		−0.09	0.399		−0.03	0.802		−0.15	0.092		−0.03	0.677
**Boys**												
*Black*												
Boys IQ^a^	793	0.06	0.369	840	0.07	0.245	846	0.04	0.420	824	0.02	0.631
Mothers IQ^b^		0.03	0.769		0.06	0.440		**0.17**	**0.030**		0.05	0.489
*Hispanic*												
Boys IQ^a^	576	0.08	0.290	552	**0.19**	**0.008**	**546**	**0.17**	**0.014**	531	0.02	0.806
Mothers IQ^b^		−0.08	0.424		−0.09	0.361		−0.07	0.448		−0.06	0.459

^a^ PIAT was the measure of offspring’s intelligence

^b^ AFQT was the measure of mothers’ intelligence

^c^ Quadratic coefficients have been italicised

*Note*: Values that are in bold are statistically significant

### Boys’ linear regression results

The bottom of Table 3 displays the results of the linear regression for non-Black and non-Hispanic boys’ BMI on their own IQ and their mothers’ IQ, with adjustment for potential confounding and mediating factors. A standard deviation increment in mothers’ IQ was negatively associated with sons’ BMI in middle childhood (beta = −0.11) in the age-adjusted analysis. This association was attenuated to the null in the fully adjusted analysis. A SD increment in boys’ IQ was positively associated with their BMI in early adolescence (beta = 0.09) in the fully adjusted model but not the baseline model (Fig. 1). There were no other significant maternal/offspring IQ-BMI associations for non-Hispanic & non-Black boys.

Some of the significant results for the Black and Hispanic boys differed from the non-Black and non-Hispanic boys’ results (Table 4 & Table S5). For boys in the Black ethnic group, a standard deviation increment in mothers’ IQ was positively associated with sons’ BMI in late childhood and across adolescence in the age-adjusted analyses. These associations were attenuated in the fully adjusted analyses and only remained significant in early adolescence (beta = 0.17; Fig. 1). For boys in the Hispanic group, a standard deviation increment in boys’ IQ was positively associated with their BMI in late childhood in the age-adjusted analysis. In the fully adjusted analysis, the association in late childhood increased in size (beta = 0.19) and the association in early adolescence became significant (beta = 0.17; Fig. 1).

In the analyses described above, we did not examine the independent role played by family SES and pre-pregnancy BMI in the maternal/offspring IQ-BMI association. It is unlikely that pre-pregnancy BMI mediated the relationship between mothers’ IQ and offspring BMI because in this sample pre-pregnancy BMI was not associated with mothers’ early life IQ.

There was essentially no evidence of an interaction between maternal/offspring IQ and family SES on offspring’s BMI (data not shown).

We repeated the linear regression analyses including adjustment for birth weight, gestation and infant feeding but including these covariates had little or no impact on associations between maternal IQ and offspring BMI (data not shown).

### Multinomial regression results

The results of the multinomial regression analyses of the association between IQ and BMI category are shown in supplementary Table S6 (girls) and supplementary Table S7 (boys). The results are generally in agreement with the linear regression analyses. In the fully adjusted model, a SD increment in maternal IQ was associated with lower odds of having obese daughters for the non-Black and non-Hispanic ethnic group in each age group except for late childhood. The odds ratios ranged from 0.59 (95% CI 0.41–0.83, *p* = 0.003) in early adolescence to 0.61 (95% CI 0.44–0.84, *p* = 0.003) in middle childhood, in the fully adjusted model (Table S6). For the black ethnic group, a standard deviation increment in mothers’ IQ was associated with increased odds of having an overweight son (OR = 1.35, 95% CI 1.01–1.80, *p* = 0.046) in early adolescence after adjusting for covariates (Table S7).

## Discussion

We examined the associations between maternal and offspring IQ and offspring BMI in youth. Looking first at girls, higher maternal IQ was associated with lower BMI in all age groups in the non-Black and non-Hispanic ethnic group. By contrast, there was no association between maternal IQ and offspring BMI in the Hispanic group after adjustment for covariates, and in the Black ethnic group, associations were curvilinear and only present in childhood. The association between girls’ IQ and BMI was curvilinear in the Black ethnic group, across adolescence, and in the non-Black and non-Hispanic ethnic group, in middle childhood. There were no associations between girls’ IQ and BMI in the Hispanic ethnic group, after adjusting for covariates.

Looking at the results for the boys, higher maternal intelligence was associated with higher BMI in the Black ethnic group, in early adolescence, after adjusting for covariates. There were no associations between maternal IQ and sons’ BMI in the Hispanic ethnic group or in the non-Black and non-Hispanic ethnic group in the fully adjusted analyses. Upon adjustment for the study covariates, higher offspring IQ was associated with higher BMI for boys in the non-Black/non-Hispanic group in early adolescence and for boys in the Hispanic ethnic group in late childhood and early adolescence. Boys’ IQ was not associated with their BMI in the Black ethnic group.

Many of the results from the linear regressions were also observed in the multinomial logistic regressions; however, there were some results that were present in the latter but not the former. The observed discrepancies could be due low power across certain BMI categories.

The observed trans-generational maternal IQ offspring BMI association was novel to this study. The positive IQ-BMI association for boys and the curvilinear association for girls were also findings that were novel to the present study. These observations were not consistently found across age group and ethnicity.

The heterogeneous relationships between maternal/offspring IQ and offspring BMI that were found across ethnicity and sex could be due to ethnic differences in the values underlying diet, exercise, and gender role, and whether or not being overweight or obese is thought to be associated with adverse health effects [39]. Ideal body shape is known to vary across ethnic group and sex. For instances the ideal body shape for Black women is larger than it is for non-Hispanic white women [39, 48]. The observed offspring/maternal IQ and BMI relationships could be explained by offspring achieving the body shape that is valued for their sex and by their ethnic group, via their own or their mothers’ intelligence [39, 48]. More intelligent mothers’ and more intelligent children could have higher health literacy, which helps them to achieve their ideal BMI [39, 48]. This line of reasoning is partly supported by studies that have found health literacy to be related to adolescent BMI [49] and other work, which has conceptualized health literacy as a specialized domain of cognitive ability [50].

The association between maternal/offspring IQ and BMI could also be partly due to genetic factors. Marioni et al. [10]. found seven genetic variants that were associated with BMI and intelligence, correlations ranged from −0.51 to −0.10. This would probably only explain some of the observed negative IQ-BMI associations.

### Strengths and limitations

This present study had several strengths. First, the IQ-BMI associations were analyzed at four different stages of youth instead of at a single year of age, which previous studies have done [5, 12–15, 17, 18]. Second, the associations of interest were analyzed in girls and boys separately, which was done in some [12, 15, 19] but not in other previous studies [5, 13, 16–18]. Third, we analysed the IQ–BMI association separately by ethnic group categories, where as other studies only adjusted for ethnicity [5, 17, 20]. This was valuable as it demonstrated that the IQ–BMI association varied by ethnic group. Fourth, this study included continuous measures of intelligence and BMI as well as information on BMI by category. Several previous studies only treated intelligence and/or BMI as a categorical variable [7, 13, 12, 16]. Fifth, the analyses were based on the NLSY, which is a large inter-generational sample of mothers and their children that are representative of the American population.

The present study had some limitations. First, it did not include a measure of fathers’ IQ or education. Second, BMI is limited as a measure of adiposity because it does not distinguish between fat and lean muscle mass [51]. Third, mothers’ pre-pregnancy BMI and, in many cases, offspring’s BMI was derived from mothers’ self-reports of height and weight. Mothers tend to overestimate the BMI of their children who are under the age of 12 and underestimate the BMI of their older children [52, 53]. Due to low numbers, it was not possible to restrict the analyses to those whose BMI was derived from measured height and weight (Table S1). It is unlikely that self-reports of maternal pre-pregnancy BMI biased the results because these tend to be valid reports of BMI [54]. Mothers may also have been biased towards reporting more socially desirable height and weight for their children based on their ethnicity and the child’s sex [55]. While this might bias ethnic differences in BMI, it is less likely to bias the association of IQ with BMI. Fourth, physical activity was not included as a covariate as it was not measured consistently across all survey years. Fifth, due to the large amount of missing data we did not include measures of offspring’s mental health as a covariate.

In conclusion, the findings from this study suggest that both mothers’ intelligence and offspring’s intelligence may lie on the pathway that influences BMI in youth but the strength and direction of the associations vary by sex, ethnicity and age group. Future studies could investigate the factors that underlie the observed differences in the IQ–childhood BMI relationship. Childhood obesity interventions may benefit from acknowledging the heterogeneous influence that intelligence has on childhood BMI.

## Electronic supplementary material

Summary of Supplementary information

Table S1

Table S2

Table S3

Table S4

Table S5

Table S6

Table S7
