# Supplementary material for: Maternal and offspring intelligence in relation to BMI across childhood and adolescence
Source: Int J Obes (Lond). 2018 Jan 30;42(9):1610–20. doi: 10.1038/s41366-018-0009-1 (PMC6002784; doi:10.1038/s41366-018-0009-1)
Supplement: Supplementary file 1 — Summary of Supplementary information [file 41366_2018_9_MOESM1_ESM.docx]

Supplementary Information TableS1

*Text summary:* Table S1 contains the number of boys and girls who had their height and weight measured, recalled by their mother, or did not report how their height and weight was reported, at interview, across age groups.

*File format*: .docx

Supplementary Information TableS2

*Text summary:* Table S2 contains the distribution of boys and girls by BMI category (under weight, healthy weight, over weight, and obese) across ethnicity and age group.

*File format*: .docx

Supplementary Information TableS3

*Text summary:* Table S3 contains the correlation matrix for the girls’ outcome variables, explanatory variables, and covariates across childhood and adolescence.

*File format*: .docx

Supplementary Information TableS4

*Text summary:* Table S4 contains the correlation matrix for the boys’ outcome variables, explanatory variables, and covariates across childhood and adolescence.

*File format*: .docx

Supplementary Information TableS5

*Text summary:* Table 5S contains the full results from the linear regression analyses of the relation between an SD increase in IQ and Black and Hispanic girls’ and boys’ BMI in childhood and adolescence adjusting for potential confounding and/or mediating variables

*File format*: .docx

Supplementary Information TableS6

*Text summary:* This table contains the results from the multinomial logistic regression analyses of the relation between an SD increase in IQ and girl’s BMI category across childhood and adolescence adjusting for potential confounding and/or mediating variables.

*File format*: .docx

Supplementary Information TableS7

*Text summary:* This table contains the results from the multinomial logistic regression analyses of the relation between an SD increase in IQ and boy’s BMI category across childhood and adolescence adjusting for potential confounding and/or mediating variables.

*File format*: .docx
