# Supplementary material for: Maternal and offspring intelligence in relation to BMI across childhood and adolescence
Source: Int J Obes (Lond). 2018 Jan 30;42(9):1610–20. doi: 10.1038/s41366-018-0009-1 (PMC6002784; doi:10.1038/s41366-018-0009-1)
Supplement: Supplementary file 2 — Table S1 [file 41366_2018_9_MOESM2_ESM.docx]

Table S1

Observations across method for reporting BMI information at interview

(measured, recalled, or method not reported) for boys and girls across

age groups.

| BMI recorded | | Boys | | Girls | |
| --- | --- | --- | --- | --- | --- |
|  |  | N | % | N | % |
| Middle childhood | Recalled | 806 | 14 | 789 | 14 |
|  | Measured | 2,925 | 50 | 2,814 | 50 |
|  | Method not reported | 2,145 | 36 | 2,032 | 36 |
|  |  |  |  |  |  |
| Late childhood | Recalled | 989 | 17 | 948 | 17 |
|  | Measured | 2,623 | 45 | 2,580 | 46 |
|  | Method not reported | 2,264 | 38 | 2,107 | 37 |
|  |  |  |  |  |  |
| Early Adolescence | Recalled | 1,326 | 23 | 1,359 | 24 |
|  | Measured | 1,909 | 32 | 1,750 | 31 |
|  | Method not reported | 2,641 | 45 | 2,526 | 45 |
|  |  |  |  |  |  |
| Middle Adolescence | Recalled | 3,575 | 61 | 3,391 | 60 |
|  | Measured | 369 | 6 | 387 | 7 |
|  | Method not reported | 1,932 | 33 | 1,857 | 33 |
|  |  |  |  |  |  |
