# Supplementary material for: Maternal and offspring intelligence in relation to BMI across childhood and adolescence
Source: Int J Obes (Lond). 2018 Jan 30;42(9):1610–20. doi: 10.1038/s41366-018-0009-1 (PMC6002784; doi:10.1038/s41366-018-0009-1)
Supplement: Supplementary file 3 — Table S2 [file 41366_2018_9_MOESM3_ESM.docx]

Table S2

Distribution of boys and girls by BMI category across ethnicity and age group.

| Sex | Age group | BMI Category | Ethnic group n (%) | | | |
| --- | --- | --- | --- | --- | --- | --- |
|  |  |  | All ethnic groups | non-Black & non-Hispanic | Black | Hispanic |
| Girls | Middle childhood | Under weight | 575 (15) | 307 (16) | 152 (13) | 116 (15) |
|  |  | Healthy weight | 2 322 (60) | 1 239 (64) | 627 (56) | 455 (58) |
|  |  | Over weight | 556 (14) | 261 (13) | 179 (16) | 116 (15) |
|  |  | Obese | 403 (11) | 139 (7) | 169 (15) | 94 (12) |
|  |  |  |  |  |  |  |
|  | Late childhood | Under weight | 482 (12) | 245 (13) | 128 (10) | 109 (14) |
|  |  | Healthy weight | 2 206 (55) | 1 159 (60) | 626(51) | 421 (53) |
|  |  | Over weight | 828 (21) | 382 (20) | 272 (22) | 172 (21) |
|  |  | Obese | 465 (12) | 158 (8) | 207 (17) | 100 (12) |
|  |  |  |  |  |  |  |
|  | Early adolescence | Under weight | 301 (8) | 164 (9) | 77 (6) | 60 (8) |
|  |  | Healthy weight | 2 210 (58) | 1 109 (63) | 643 (52) | 458 (57) |
|  |  | Over weight | 865 (23) | 364 (21) | 304 (24) | 196 (24) |
|  |  | Obese | 435 (11) | 125 (7) | 219 (18) | 91 (11) |
|  |  |  |  |  |  |  |
|  | Middle adolescence | Under weight | 248 (7) | 141 (8) | 56 (5) | 51 (7) |
|  |  | Healthy weight | 2 293 (61) | 1 154 (67) | 654 (52) | 485 (61) |
|  |  | Over weight | 752 (20) | 260 (15) | 317 (25) | 174 (22) |
|  |  | Obese | 467 (12) | 156 (9) | 232 (18) | 79 (10) |
|  |  |  |  |  |  |  |
| Boys | Middle childhood | Under weight | 654 (16) | 337 (16) | 177 (15) | 140 (16) |
|  |  | Healthy weight | 2 564 (62) | 1 358 (65) | 702 (61) | 503 (59) |
|  |  | Over weight | 515 (13) | 245 (12) | 156 (13) | 114 (13) |
|  |  | Obese | 369 (9) | 145 (7) | 122 (11) | 102 (12) |
|  |  |  |  |  |  |  |
|  | Late childhood | Under weight | 413 (10) | 206 (10) | 122 (10) | 85 (10) |
|  |  | Healthy weight | 2 476 (61) | 1 239 (63) | 756 (61) | 481 (56) |
|  |  | Over weight | 759 (19) | 354 (18) | 213 (17) | 192 (22) |
|  |  | Obese | 439 (11) | 181 (9) | 151 (12) | 107 (12) |
|  |  |  |  |  |  |  |
|  | Early adolescence | Under weight | 252 (7) | 110 (6) | 82 (7) | 60 (7) |
|  |  | Healthy weight | 2 321 (60) | 1 138 (63) | 721 (58) | 462 (55) |
|  |  | Over weight | 830 (21) | 359 (20) | 263 (21) | 208 (25) |
|  |  | Obese | 476 (12) | 194 (11) | 168 (14) | 114 (14) |
|  |  |  |  |  |  |  |
|  | Middle adolescence | Under weight | 134 (3) | 56 (3) | 36 (3) | 42 (5) |
|  |  | Healthy weight | 2 419 (62) | 1 146 (64) | 778 (61) | 495 (57) |
|  |  | Over weight | 859 (22) | 368 (21) | 292 (23) | 199 (23) |
|  |  | Obese | 519 (13) | 22 (12) | 162 (13) | 135 (15) |
