# Supplementary material for: Maternal and offspring intelligence in relation to BMI across childhood and adolescence
Source: Int J Obes (Lond). 2018 Jan 30;42(9):1610–20. doi: 10.1038/s41366-018-0009-1 (PMC6002784; doi:10.1038/s41366-018-0009-1)
Supplement: Supplementary file 4 — Table S3 [file 41366_2018_9_MOESM4_ESM.docx]

Table S3

Correlation matrix for outcome variables, explanatory variables, and covariates across childhood and adolescence for girl’s

|  | | Mothers AFQT | Children's PIAT | | | Pre pregnancy BMI | Children's BMI ^a^ | | | | Family SES | | | |
| --- | --- | --- | --- | --- | --- | --- | --- | --- | --- | --- | --- | --- | --- | --- |
|  |  |  | Middle childhood | Late childhood | Early adolescence |  | Middle childhood | Late childhood | Early adolescence | Middle adolescence | Middle childhood | Late childhood | Early adolescence | Middle adolescence |
|  | Mothers AFQT | 1.00 |  |  |  |  |  |  |  |  |  |  |  |  |
|  |  |  |  |  |  |  |  |  |  |  |  |  |  |  |
|  |  | 4 490 |  |  |  |  |  |  |  |  |  |  |  |  |
| Children's PIAT | Middle childhood | 0.444 | 1.00 |  |  |  |  |  |  |  |  |  |  |  |
|  |  | <0.001 |  |  |  |  |  |  |  |  |  |  |  |  |
|  |  | 3 638 | 3 780 |  |  |  |  |  |  |  |  |  |  |  |
|  | Late childhood | 0.498 | 0.708 | 1.00 |  |  |  |  |  |  |  |  |  |  |
|  |  | <0.001 | <0.001 |  |  |  |  |  |  |  |  |  |  |  |
|  |  | 3 661 | 3 256 | 3 800 |  |  |  |  |  |  |  |  |  |  |
|  | Early adolescence | 0.521 | 0.661 | 0.817 | 1.00 |  |  |  |  |  |  |  |  |  |
|  |  | <0.001 | <0.001 | <0.001 |  |  |  |  |  |  |  |  |  |  |
|  |  | 3 466 | 3 021 | 3 262 | 3 605 |  |  |  |  |  |  |  |  |  |
|  | Pre Pregnancy BMI | -0.015 | -0.036 | -0.033 | -0.036 | 1.00 |  |  |  |  |  |  |  |  |
|  |  | 0.362 | 0.040 | 0.060 | 0.042 |  |  |  |  |  |  |  |  |  |
|  |  | 3 899 | 3 353 | 3 357 | 3 171 | 4 061 |  |  |  |  |  |  |  |  |
| Children's BMI ^a^ | Middle childhood | -0.073 | -0.019 | -0.034 | -0.046 | 0.249 | 1.00 |  |  |  |  |  |  |  |
|  |  | <0.001 | 0.253 | 0.053 | 0.012 | <0.001 |  |  |  |  |  |  |  |  |
|  |  | 3 690 | 3 647 | 3 260 | 3 018 | 3 400 | 3 855 |  |  |  |  |  |  |  |
|  | Late childhood | -0.084 | -0.035 | -0.029 | -0.049 | 0.305 | 0.579 | 1.00 |  |  |  |  |  |  |
|  |  | <0.001 | 0.041 | 0.077 | 0.005 | <0.001 | <0.001 |  |  |  |  |  |  |  |
|  |  | 3 822 | 3 330 | 3 720 | 3 319 | 3 488 | 3 372 | 3 981 |  |  |  |  |  |  |
|  | Early adolescence | -0.148 | -0.075 | -0.085 | -0.101 | 0.322 | 0.543 | 0.725 | 1.00 |  |  |  |  |  |
|  |  | <0.001 | <0.001 | <0.001 | <0.001 | <0.001 | <0.001 | <0.001 |  |  |  |  |  |  |
|  |  | 3 652 | 3 135 | 3 375 | 3 566 | 3 328 | 3 156 | 3 478 | 3 811 |  |  |  |  |  |
|  | Middle adolescence | -0.185 | -0.119 | -0.143 | -0.161 | 0.306 | 0.509 | 0.634 | 0.746 | 1.00 |  |  |  |  |
|  |  | <0.001 | <0.001 | <0.001 | <0.001 | <0.001 | <0.001 | <0.001 | <0.001 |  |  |  |  |  |
|  |  | 3 616 | 3 119 | 3 301 | 3 283 | 3 318 | 3 141 | 3 408 | 3 438 | 3 774 |  |  |  |  |
| Family  SES | Middle childhood | 0.652 | 0.397 | 0.428 | 0.434 | 0.034 | -0.011 | -0.048 | -0.125 | -0.157 | 1.00 |  |  |  |
|  |  | <0.001 | <0.001 | <0.001 | <0.001 | 0.059 | 0.527 | 0.007 | <0.001 | <0.001 |  |  |  |  |
|  |  | 3 419 | 3 356 | 3 019 | 2 825 | 3 153 | 3 397 | 3 123 | 2 954 | 2 922 | 3560 |  |  |  |
|  | Late childhood | 0.657 | 0.401 | 0.420 | 0.431 | 0.052 | -0.020 | -0.022 | -0.108 | -0.154 | 0.913 | 1.00 |  |  |
|  |  | <0.001 | <0.001 | <0.001 | <0.001 | 0.004 | 0.264 | 0.184 | <0.001 | <0.001 | <0.001 |  |  |  |
|  |  | 3 487 | 3 041 | 3 372 | 3 031 | 3 192 | 3 075 | 3 531 | 3 182 | 3 122 | 2 913 | 3 624 |  |  |
|  | Early adolescence | 0.658 | 0.409 | 0.418 | 0.438 | 0.048 | -0.002 | -0.004 | -0.084 | -0.125 | 0.883 | 0.914 | 1.00 |  |
|  |  | <0.001 | <0.001 | <0.001 | <0.001 | 0.009 | 0.911 | 0.832 | <0.001 | <0.001 | <0.001 | <0.001 |  |  |
|  |  | 3 242 | 2 795 | 3 004 | 3 160 | 2 970 | 2 816 | 3 086 | 3 309 | 3 051 | 2 682 | 2 898 | 3 373 |  |
|  | Middle adolescence | 0.664 | 0.400 | 0.416 | 0.433 | -0.002 | -0.027 | -0.035 | -0.100 | -0.139 | 0.841 | 0.869 | 0.896 | 1.00 |
|  |  | <0.001 | <0.001 | <0.001 | <0.001 | 0.901 | 0.160 | 0.056 | <0.001 | <0.001 | <0.001 | <0.001 | <0.001 |  |
|  |  | 3 200 | 2 791 | 2 943 | 2 946 | 2 945 | 2 809 | 3 020 | 3 069 | 3 296 | 2 652 | 2 805 | 2 782 | 3 320 |

Note. ^a^ BMI scores have been z-anthro transformed against the WHO 2007 growth charts.
