# Supplementary material for: Maternal and offspring intelligence in relation to BMI across childhood and adolescence
Source: Int J Obes (Lond). 2018 Jan 30;42(9):1610–20. doi: 10.1038/s41366-018-0009-1 (PMC6002784; doi:10.1038/s41366-018-0009-1)
Supplement: Supplementary file 5 — Table S4 [file 41366_2018_9_MOESM5_ESM.docx]

Table S4

Correlation matrix for outcome variables, explanatory variables, and covariates across childhood and adolescence for boy’s

|  | | Mothers AFQT | Children's PIAT | | | Pre pregnancy BMI | Children's BMI ^a^ | | | | Family SES | | | |
| --- | --- | --- | --- | --- | --- | --- | --- | --- | --- | --- | --- | --- | --- | --- |
|  |  |  | Middle childhood | Late childhood | Early adolescence |  | Middle childhood | Late childhood | Early adolescence | Middle adolescence | Middle childhood | Late childhood | Early adolescence | Middle adolescence |
|  | Mothers AFQT | 1.00 |  |  |  |  |  |  |  |  |  |  |  |  |
|  |  |  |  |  |  |  |  |  |  |  |  |  |  |  |
|  |  | 4 726 |  |  |  |  |  |  |  |  |  |  |  |  |
| Children's PIAT | Middle childhood | 0.448 | 1.00 |  |  |  |  |  |  |  |  |  |  |  |
|  |  | <0.001 |  |  |  |  |  |  |  |  |  |  |  |  |
|  |  | 3 796 | 3 919 |  |  |  |  |  |  |  |  |  |  |  |
|  | Late childhood | 0.498 | 0.725 | 1.00 |  |  |  |  |  |  |  |  |  |  |
|  |  | <0.001 | <0.001 |  |  |  |  |  |  |  |  |  |  |  |
|  |  | 3 691 | 3 266 | 3 816 |  |  |  |  |  |  |  |  |  |  |
|  | Early adolescence | 0.525 | 0.684 | 0.854 | 1.00 |  |  |  |  |  |  |  |  |  |
|  |  | <0.001 | <0.001 | <0.001 |  |  |  |  |  |  |  |  |  |  |
|  |  | 3 488 | 3 009 | 3 187 | 3 611 |  |  |  |  |  |  |  |  |  |
|  | Pre Pregnancy BMI | -0.015 | -0.065 | -0.083 | -0.071 | 1.00 |  |  |  |  |  |  |  |  |
|  |  | 0.345 | 0.001 | <0.001 | <0.001 |  |  |  |  |  |  |  |  |  |
|  |  | 4 114 | 3 454 | 3 359 | 3 210 | 4 275 |  |  |  |  |  |  |  |  |
| Children's BMI ^a^ | Middle childhood | -0.063 | -0.016 | -0.021 | -0.036 | 0.212 | 1.00 |  |  |  |  |  |  |  |
|  |  | 0.001 | 0.326 | 0.231 | 0.046 | <0.001 |  |  |  |  |  |  |  |  |
|  |  | 3 931 | 3 819 | 3 308 | 3 060 | 3 577 | 4 079 |  |  |  |  |  |  |  |
|  | Late childhood | -0.026 | -0.004 | 0.007 | 0.017 | 0.286 | 0.529 | 1.00 |  |  |  |  |  |  |
|  |  | 0.104 | 0.824 | 0.685 | 0.337 | <0.001 | <0.001 |  |  |  |  |  |  |  |
|  |  | 3 931 | 3 411 | 3 763 | 3 308 | 3 579 | 3 505 | 4 087 |  |  |  |  |  |  |
|  | Early adolescence | -0.018 | 0.0004 | 0.009 | 0.013 | 0.290 | 0.510 | 0.697 | 1.00 |  |  |  |  |  |
|  |  | 0.263 | 0.981 | 0.622 | 0.455 | <0.001 | <0.001 | <0.001 |  |  |  |  |  |  |
|  |  | 3 727 | 3 169 | 3 333 | 3 574 | 3 422 | 3 265 | 3 516 | 3 879 |  |  |  |  |  |
|  | Middle adolescence | -0.038 | -0.027 | -0.022 | -0.031 | 0.281 | 0.495 | 0.613 | 0.699 | 1.00 |  |  |  |  |
|  |  | 0.020 | 0.128 | 0.205 | 0.072 | <0.001 | <0.001 | <0.001 | <0.001 |  |  |  |  |  |
|  |  | 3 784 | 3 200 | 3 289 | 3 310 | 3 475 | 3 303 | 3 475 | 3 503 | 3 941 |  |  |  |  |
| Family  SES | Middle childhood | 0.643 | 0.407 | 0.438 | 0.467 | 0.030 | -0.017 | 0.001 | -0.007 | -0.022 | 1.00 |  |  |  |
|  |  | <0.001 | <0.001 | <0.001 | <0.001 | 0.086 | 0.322 | 0.935 | 0.707 | 0.228 |  |  |  |  |
|  |  | 3 578 | 3 457 | 3 016 | 2 799 | 3 277 | 3 571 | 3 197 | 2980 | 3023 | 3710 |  |  |  |
|  | Late childhood | 0.649 | 0.395 | 0.434 | 0.461 | 0.028 | -0.033 | 0.009 | 0.008 | -0.014 | 0.914 | 1.00 |  |  |
|  |  | <0.001 | <0.001 | <0.001 | <0.001 | 0.111 | 0.068 | 0.577 | 0.655 | 0.444 | <0.001 |  |  |  |
|  |  | 3 518 | 3 049 | 3 340 | 2 943 | 3 215 | 3 133 | 3 554 | 3 134 | 3 099 | 2 921 | 3 651 |  |  |
|  | Early adolescence | 0.655 | 0.418 | 0.436 | 0.457 | 0.029 | -0.034 | 0.002 | 0.005 | -0.033 | 0.877 | 0.907 | 1.00 |  |
|  |  | <0.001 | <0.001 | <0.001 | <0.001 | 0.115 | 0.064 | 0.907 | 0.418 | 0.034 | <0.001 | <0.001 |  |  |
|  |  | 3 317 | 2 828 | 2 959 | 3 163 | 3 055 | 2 908 | 3 132 | 3 370 | 3 122 | 2 716 | 2 864 | 3 443 |  |
|  | Middle adolescence | 0.649 | 0.398 | 0.433 | 0.455 | -0.029 | -0.048 | -0.008 | -0.012 | -0.030 | 0.842 | 0.861 | 0.895 | 1.00 |
|  |  | <0.001 | <0.001 | <0.001 | <0.001 | 0.112 | 0.010 | 0.664 | 0.503 | 0.084 | <0.001 | <0.001 | <0.001 |  |
|  |  | 3 283 | 2 821 | 2 907 | 2 938 | 3 016 | 2 896 | 3 054 | 3 091 | 3 388 | 2 700 | 2 768 | 2 820 | 3 404 |

Note. ^a^ BMI scores have been z-anthro transformed against the WHO 2007 growth charts.
