# Supplementary material for: Maternal and offspring intelligence in relation to BMI across childhood and adolescence
Source: Int J Obes (Lond). 2018 Jan 30;42(9):1610–20. doi: 10.1038/s41366-018-0009-1 (PMC6002784; doi:10.1038/s41366-018-0009-1)
Supplement: Supplementary file 6 — Table S5 [file 41366_2018_9_MOESM6_ESM.docx]

| Table S5 |  |  |  |  | |  | |  | |  | |  | |  | |  |  | |  |
| --- | --- | --- | --- | --- | --- | --- | --- | --- | --- | --- | --- | --- | --- | --- | --- | --- | --- | --- | --- |
| Regression analyses of the relation between an SD increase in IQ and Black and Hispanic girls’ and boys’ BMI in childhood and adolescence adjusting for potential confounding and/or mediating variables. | | | | | | | | | | | | | | | | | | | |
|  | Middle Childhood | | | Late Childhood | | | | | Early Adolescence | | | | | | Middle Adolescence | | | | |
|  |  | | |  | | | | |  | | | | | |  | | | | |
|  | N | Beta (95% CI) | P value | N | Beta (95% CI) | | P value | | N | | Beta (95% CI) | | P value | | N | | Beta (95% CI) | P value | |
| **Girls** |  |  |  |  |  | |  | |  | |  | |  | |  | |  |  | |
| **Black ^d^** |  |  |  |  |  | |  | |  | |  | |  | |  | |  |  | |
| Girls IQ |  |  |  |  |  | |  | |  | |  | |  | |  | |  |  | |
| Baseline model | 819 | -0.05 (-0.16 to 0.05) | 0.302 | 886 | -0.02 (-0.13 to 0.08) | | 0.642 | | 863 | | **-0.11 (-0.22 to -0.000)** | | **0.051** | | 852 | | **-0.25 (-0.35 to -0.15)** | **<0.001** | |
| *Quadratic coefficient* |  |  |  |  |  | |  | |  | | ***-0.09 (-0.17 to -0.02)*** | | ***0.013*** | |  | | ***-0.07 (-0.14 to -0.01)*** | ***0.031*** | |
| Fully adjusted model |  | -0.05 (-0.16 to 0.06) | 0.393 |  | -0.04 (-0.15 to 0.07) | | 0.445 | |  | | **-0.12 (-0.24 to -0.004)** | | **0.043** | |  | | **-0.22 (-0.33 to -0.12)** | **<0.001** | |
| *Quadratic coefficient* |  |  |  |  |  | |  | |  | | ***-0.08 (-0.15 to -0.01)*** | | ***0.023*** | |  | | ***-0.07 (-0.13 to -0.004)*** | ***0.038*** | |
| Mothers IQ |  |  |  |  |  | |  | |  | |  | |  | |  | |  |  | |
| Baseline model |  | **-0.21 (-0.47 to 0.06)** | **0.130** |  | **-0.18 (-0.45 to 0.08)** | | **0.172** | |  | | 0.06 (-0.05 to 0.18) | | 0.292 | |  | | -0.09 (-0.20 to 0.01) | 0.090 | |
| *Quadratic coefficient* |  | ***-0.14 (-0.27 to -0.01)*** | ***0.034*** |  | ***-0.16 (-0.29 to -0.04)*** | | ***0.009*** | |  | |  | |  | |  | |  |  | |
| Fully adjusted model |  | **-0.20 (-0.49 to 0.09)** | **0.182** |  | **-0.25 (-0.52 to 0.03)** | | **0.078** | |  | | 0.06 (-0.09 to 0.21) | | 0.438 | |  | | -0.04 (-0.17 to 0.10) | 0.610 | |
| *Quadratic coefficient* |  | ***-0.17 (-0.30 to -0.04)*** | ***0.011*** |  | ***-0.20 (-0.31 to -0.08)*** | | ***0.001*** | |  | |  | |  | |  | |  |  | |
| **Hispanic** |  |  |  |  |  | |  | |  | |  | |  | |  | |  |  | |
| Girls IQ |  |  |  |  |  | |  | |  | |  | |  | |  | |  |  | |
| Baseline model | 507 | -0.04 (-0.16 to 0.08) | 0.536 | 534 | 0.05 (-0.08 to 0.17) | | 0.438 | | 521 | | -0.09 (-0.21 to 0.02) | | 0.096 | | 480 | | **-0.12 (-0.22 to -0.02)** | **0.024** | |
| Fully adjusted model |  | 0.03 (-0.10 to 0.17) | 0.632 |  | 0.10 (-0.03 to 0.22) | | 0.143 | |  | | -0.02 (-0.13 to 0.10) | | 0.754 | |  | | -0.07 (-0.18 to 0.04) | 0.213 | |
| Mothers IQ |  |  |  |  |  | |  | |  | |  | |  | |  | |  |  | |
| Baseline model |  | -0.08 (-0.23 to 0.08) | 0.338 |  | -0.05 (-0.20 to 0.11) | | 0.563 | |  | | **-0.18 (-0.32 to -0.05)** | | **0.009** | |  | | **-0.13 (-0.25 to -0.001)** | **0.051** | |
| Fully adjusted model |  | -0.09 (-0.29 to 0.12) | 0.399 |  | -0.03 (-0.22 to 0.17) | | 0.802 | |  | | -0.15 (-0.32 to 0.02) | | 0.092 | |  | | -0.03 (-0.20 to 0.13) | 0.677 | |
|  |  |  |  |  |  | |  | |  | |  | |  | |  | |  |  | |
| **Boys** |  |  |  |  |  | |  | |  | |  | |  | |  | |  |  | |
| **Black** |  |  |  |  |  | |  | |  | |  | |  | |  | |  |  | |
| Boys IQ |  |  |  |  |  | |  | |  | |  | |  | |  | |  |  | |
| Baseline model | 793 | 0.06 (-0.05 to 0.18) | 0.267 | 840 | 0.08 (-0.02 to 0.19) | | 0.116 | | 846 | | 0.09 (-0.01 to 0.19) | | 0.083 | | 824 | | 0.02 (-0.07 to 0.11) | 0.632 | |
| Fully adjusted model |  | 0.06 (-0.07 to 0.18) | 0.369 |  | 0.07 (-0.04 to 0.17) | | 0.245 | |  | | 0.04 (-0.06 to 0.15) | | 0.420 | |  | | 0.02 (-0.07 to 0.12) | 0.631 | |
|  |  |  |  |  |  | |  | |  | |  | |  | |  | |  |  | |
| Mothers IQ |  |  |  |  |  | |  | |  | |  | |  | |  | |  |  | |
| Baseline model |  | 0.12 (-0.02 to 0.26) | 0.102 |  | **0.16 (0.03 to 0.30)** | | **0.019** | |  | | **0.21 (0.09 to 0.34)** | | **0.001** | |  | | **0.11 (0.002 to 0.22**) | **0.045** | |
| Fully adjusted model |  | 0.03 (-0.15 to 0.20) | 0.769 |  | 0.06 (-0.10 to 0.23) | | 0.440 | |  | | **0.17 (0.02 to 0.32)** | | **0.030** | |  | | 0.05 (-0.09 to 0.19) | 0.489 | |
|  |  |  |  |  |  | |  | |  | |  | |  | |  | |  |  | |
| **Hispanic** |  |  |  |  |  | |  | |  | |  | |  | |  | |  |  | |
| Boys IQ |  |  |  |  |  | |  | |  | |  | |  | |  | |  |  | |
| Baseline model | 576 | 0.05 (-0.09 to 0.19) | 0.474 | 552 | **0.14 (0.01 to 0.27)** | | **0.038** | | 546 | | 0.09 (-0.04 to 0.21) | | 0.193 | | 531 | | -0.05 (-0.16 to 0.07) | 0.405 | |
| Fully adjusted model |  | 0.08 (-0.07 to 0.23) | 0.290 |  | **0.19 (0.05 to 0.33)** | | **0.008** | |  | | **0.17 (0.04 to 0.31)** | | **0.014** | |  | | 0.02 (-0.11 to 0.14) | 0.806 | |
|  |  |  |  |  |  | |  | |  | |  | |  | |  | |  |  | |
| Mothers IQ |  |  |  |  |  | |  | |  | |  | |  | |  | |  |  | |
| Baseline model |  | -0.07 (-0.24 to 0.09) | 0.370 |  | -0.04 (-0.20 to 0.11) | | 0.551 | |  | | -0.05 (-0.19 to 0.10) | | 0.531 | |  | | -0.05 (-0.18 to 0.08) | 0.464 | |
| Fully adjusted model |  | -0.08 (-0.30 to 0.12) | 0.424 |  | -0.09 (-0.29 to 0.11) | | 0.361 | |  | | -0.07 (-0.26 to 0.12) | | 0.448 | |  | | -0.06 (-0.23 to 0.10) | 0.459 | |
| *Note*. ^a^ PIAT was the measure of offspring’s intelligence | | | | | | | | | | | | | | | | | | | |
| ^b^ AFQT was the measure of mothers’ intelligence  ^*^ values that are in **bold** are statistically significant. | | | | | | | | | | | | | | | | | | | |
| Baseline Model: PIAT or AFQT & child age  Fully adjusted Model: PIAT, AFQT, child age, mothers' pre-pregnancy BMI, & family SES (net family income, year income was recorded, & maternal education). | | | | | | | | | | | | | | | | | | | |
