# Supplementary material for: Maternal and offspring intelligence in relation to BMI across childhood and adolescence
Source: Int J Obes (Lond). 2018 Jan 30;42(9):1610–20. doi: 10.1038/s41366-018-0009-1 (PMC6002784; doi:10.1038/s41366-018-0009-1)
Supplement: Supplementary file 7 — Table S6 [file 41366_2018_9_MOESM7_ESM.docx]

Table S6

| Multinomial logistic regression analyses of the relation between an SD increase in IQ and girls’ BMI category across childhood and adolescence adjusting for potential confounding and/or mediating variables. | | | | | | | | | | | | | | | |  |  |  |
| --- | --- | --- | --- | --- | --- | --- | --- | --- | --- | --- | --- | --- | --- | --- | --- | --- | --- | --- |
|  | |  | Middle Childhood | | | Late Childhood | | | | Early Adolescence | | | Middle Adolescence | | |  |  |  |
|  | |  |  | Baseline model | Fully adjusted model |  | Baseline model | Fully adjusted model | |  | Baseline model | Fully adjusted model |  | Baseline model | Fully adjusted model | |  |  |
|  | |  | N | OR (95% CI), P value | OR (95% CI), P value | N | OR (95% CI), P value | OR (95% CI), P value | | N | OR (95% CI), P value | OR (95% CI), P value | N | OR (95% CI), P value | OR (95% CI), P value | |  |  |
| **Non-Black & Non-Hispanic** |  | |  |  |  |  |  | |  |  |  |  |  |  |  | |  |  |
|  |  | |  |  |  |  |  | |  |  |  |  |  |  |  | |  |  |
| Girls’ IQ | Under weight | | 221 | 0.88 (0.76 to 1.03), 0.113 | 0.89 (0.75 to 1.05), 0.170 | 172 | 1.01 (0.84 to 1.22), 0.888 | | 1.01 (0.82 to 1.24), 0.917 | 110 | 0.88 (0.70 to 1.11), 0.266 | 0.90 (0.70 to 1.15), 0.396 | 100 | **0.77 (0.61 to 0.97), 0.027** | 0.82 (0.63 to 1.07), 0.150 | |  |  |
|  | Normal | | 934 | -- | -- | 849 | -- | | -- | 815 | -- | -- | 796 | -- | -- | |  |  |
|  | Over weight | | 203 | 1.07 (0.91 to 1.26), 0.375 | 1.14 (0.95 to 1.36), 0.164 | 283 | 0.92 (0.79 to 1.07), 0.301 | | 0.90 (0.76 to 1.07), 0.239 | 274 | 0.88 (0.75 to 1.02), 0.086 | 1.04 (0.87 to 1.25), 0.658 | 179 | 0.84 (0.70 to 1.01), 0.062 | 0.92 (0.75 to 1.14), 0.465 | |  |  |
|  | Obese | | 100 | 0.97 (0.78 to 1.20), 0.761 | 1.00 (0.79 to 1.28), 0.979 | 126 | 0.92 (0.76 to 1.14), 0.463 | | 1.05 (0.83 to 1.34), 0.675 | 96 | **0.76 (0.60 to 0.96), 0.021** | 0.98 (0.75 to 1.30), 0.906 | 116 | **0.66 (0.53 to 0.81), <0.001** | 0.84 (0.65 to 1.09), 0.180 | |  |  |
|  |  | |  |  |  |  |  | |  |  |  |  |  |  |  | |  |  |
| Mothers’ IQ | Under weight | | 221 | 0.96 (0.81 to 1.14), 0.670 | 1.04 (0.83 to 1.31), 0.717 | 172 | 1.00 (0.83 to 1.21), 0.979 | | 1.04 (0.80 to 1.35), 0.779 | 110 | 0.90 (0.70 to 1.13), 0.348 | 0.97 (0.72 to 1.38), 0.981 | 100 | **0.84 (0.64 to 1.07), 0.160** | 1.13 (0.80 to 1.60), 0.481 | |  |  |
|  | Normal | | 934 | -- | -- | 849 | -- | | -- | 815 | -- | -- | 796 | **--** | -- | |  |  |
|  | Over weight | | 203 | 0.85 (0.71 to 1.02), 0.074 | **0.76 (0.59 to 0.96), 0.022** | 283 | 0.92 (0.79 to 1.08), 0.317 | | 0.94 (0.76 to 1.16), 0.600 | 274 | **0.69 (0.59 to 0.82), <0.001** | **0.76 (0.61 to 0.96), 0.018** | 179 | **0.75 (0.61 to 0.91), 0.004** | 0.84 (0.64 to 1.09), 0.193 | |  |  |
|  | Obese | | 100 | **0.71 (0.56 to 0.91), 0.007** | **0.61 (0.44 to 0.84), 0.003** | 126 | **0.69 (0.55 to 0.86), 0.001** | | 0.76 (0.51 to 0.88), 0.077 | 96 | **0.53 (0.41 to 0.68), <0.001** | **0.59 (0.41 to 0.83, 0.003** | 116 | **0.48 (0.37 to 0.61), <0.001** | **0.60 (0.44 to 0.84), 0.002** | |  |  |
|  |  | |  |  |  |  |  | |  |  |  |  |  |  |  | |  |  |
| **Black** |  | |  |  |  |  |  | |  |  |  |  |  |  |  | |  |  |
| Girls’ IQ | Under weight | | 101 | 0.88 (0.70 to 1.11), 0.276 | 0.95 (0.75 to 1.22), 0.694 | 83 | 0.95 (0.75 to 1.22), 0.697 | | 0.95 (0.73 to 1.25), 0.733 | 48 | 0.86 (0.63 to 1.17), 0.346 | 0.84 (0.59 to 1.19), 0.327 | 38 | 1.05 (0.74 to 1.51), 0.763 | 1.03 (0.70 to 1.52), 0.881 | |  |  |
|  | Normal | | 466 | -- | -- | 449 | -- | | -- | 460 | -- | -- | 453 | -- | -- | |  |  |
|  | Over weight | | 132 | 0.86 (0.71 to 1.06), 0.162 | 0.97 (0.77 to 1.22), 0.792 | 205 | 0.96 (0.81 to 1.15), 0.676 | | 0.88 (0.72 to 1.07), 0.193 | 204 | 0.94 (0.79 to 1.12), 0.480 | 0.90 (0.73 to 1.09), 0.265 | 208 | **0.79 (0.66 to 0.94), 0.008** | **0.80 (0.66 to 0.98), 0.029** | |  |  |
|  | Obese | | 120 | 0.90 (0.73 to 1.12), 0.347 | 0.94 (0.75 to 1.20), 0.639 | 149 | 0.90 (0.73 to 1.08), 0.255 | | 0.90 (0.71 to 1.14), 0.383 | 151 | 0.89 (0.73 to 1.07), 0.224 | 0.85 (0.68 to 1.07), 0.164 | 150 | **0.65 (0.53 to 0.80), <0.001** | **0.66 (0.53 to 0.84), 0.001** | |  |  |
|  |  | |  |  |  |  |  | |  |  |  |  |  |  |  | |  |  |
| Mothers’ IQ | Under weight | | 101 | **0.73 (0.54 to 0.98), 0.035** | 0.71 (0.49 to 1.04), 0.076 | 83 | 0.90 (0.66 to 1.23), 0.510 | | 0.94 (0.62 to 1.42), 0.767 | 48 | 1.01 (0.68 to 1.49), 0.962 | 1.15 (0.69 to 1.93), 0.587 | 38 | 1.08 (0.70 to 1.67), 0.726 | 1.15 (0.64 to 2.05), 0.647 | |  |  |
|  | Normal | | 466 | -- | -- | 449 | -- | | -- | 460 | -- | -- | 453 | -- | -- | |  |  |
|  | Over weight | | 132 | **0.76 (0.58 to 0.99), 0.046** | 0.90 (0.63 to 1.26), 0.514 | 205 | **1.25 (1.00 to 1.57), 0.046** | | 1.28 (0.96 to 1.72), 0.096 | 204 | 1.11 (0.90 to 1.38), 0.349 | 1.09 (0.82 to 1.46), 0.535 | 208 | 0.92 (0.74 to 1.15), 0.468 | 1.13 (0.84 to 1.51), 0.428 | |  |  |
|  | Obese | | 120 | 0.95 (0.73 to 1.26), 0.736 | 1.05 (0.73 to 1.50), 0.805 | 149 | 1.06 (0.82 to 1.36), 0.643 | | 1.17 (0.84 to 1.65), 0.347 | 151 | 1.08 (0.85 to 1.38), 0.518 | 1.11 (0.79 to 1.54), 0.549 | 150 | 0.82 (0.64 to 1.05), 0.115 | 0.94 (0.68 to 1.32), 0.744 | |  |  |
|  |  | |  |  |  |  |  | |  |  |  |  |  |  |  | |  |  |
| **Hispanic** |  | |  |  |  |  |  | |  |  |  |  |  |  |  | |  |  |
| Girls’ IQ | Under weight | | 72 | 0.97 (0.76 to 1.26), 0.835 | 0.90 (0.68 to 1.21), 0.510 | 74 | 1.04 (0.80 to 1.35), 0.753 | | 1.00 (0.75 to 1.33), 0.992 | 41 | 1.22 (0.88 to 1.72), 0.234 | 0.99 (0.66 to 1.48), 0.972 | 30 | 0.94 (0.64 to 1.39), 0.750 | 0.75 (0.48 to 1.16), 0.196 | |  |  |
|  | Normal | | 302 | -- | -- | 281 | -- | | -- | 296 | -- | -- | 288 | -- | -- | |  |  |
|  | Over weight | | 81 | 0.92 (0.73 to 1.17), 0.515 | 0.88 (0.67 to 1.15), 0.353 | 124 | **1.26 (1.01 to 1.55), 0.039** | | **1.33 (1.05 to 1.69), 0.017** | 128 | 1.11 (0.90 to 1.36), 0.366 | 1.15 (0.90 to 1.48), 0.267 | 112 | **0.74 (0.59 to 0.93), 0.010** | **0.75 (0.58 to 0.97), 0.028** | |  |  |
|  | Obese | | 52 | 0.94 (0.70 to 1.27), 0.780 | 1.17 (0.84 to 1.63), 0.344 | 55 | 1.24 (0.92 to 1.68), 0.148 | | 1.38 (0.98 to 1.92), 0.063 | 56 | 0.88 (0.66 to 1.17), 0.381 | 0.98 (0.70 to 1.36), 0.909 | 48 | 0.74 (0.54 to 1.01), 0.061 | 0.80 (0.57 to 1.14), 0.219 | |  |  |
|  |  | |  |  |  |  |  | |  |  |  |  |  |  |  | |  |  |
| Mothers’ IQ | Under weight | | 72 | 1.11 (0.79 to 1.54), 0.549 | 1.25 (0.80 to 1.95), 0.324 | 74 | 1.05 (0.76 to 1.45), 0.777 | | 0.94 (0.61 to 1.43), 0.773 | 41 | 1.43(0.96 to 2.12), 0.080 | 1.23 (0.73 to 2.12), 0.440 | 30 | 1.36 (0.87 to 2.14), 0.172 | 1.15 (0.63 to 2.10), 0.644 | |  |  |
|  | Normal | | 302 | -- | -- | 281 | -- | | -- | 296 | -- | -- | 288 | -- | -- | |  |  |
|  | Over weight | | 81 | 1.17 (0.86 to 1.60), 0.317 | 1.04 (0.68 to 1.58), 0.864 | 124 | 1.08 (0.83 to 1.42), 0.556 | | 0.99 (0.69 to 1.41), 0.963 | 128 | 1.00 (0.77 to 1.31), 0.975 | 0.84 (0.58 to 1.19), 0.313 | 112 | 0.88 (0.67 to 1.16), 0.366 | 1.04 (0.70 to 1.52), 0.857 | |  |  |
|  | Obese | | 52 | **0.64 (0.43 to 0.95), 0.029** | 0.63 (0.38 to 1.07), 0.087 | 55 | 0.98 (0.68 to 1.42), 0.931 | | 0.86 (0.52 to 1.42), 0.556 | 56 | 0.74 (0.51 to 1.07), 0.111 | 0.81 (0.48 to 1.38), 0.439 | 48 | 0.73 (0.49 to 1.07), 0.103 | 0.82 (0.47 to 1.42), 0.469 | |  |  |
| *Note*. ^a^ PIAT was the measure of girls’ intelligence | | | | | | | | | | | | | | | | | | |
| ^b^AFQT was the measure of mothers’ intelligence.  ^c^ values that are in **bold** are statistically significant.  Baseline Model: PIAT or AFQT & child age  Fully adjusted Model: PIAT, AFQT, child age, mothers' pre-pregnancy BMI, family SES (net family income, year income was recorded, & maternal education). | | | | | | | | | | | | | | | | | | |
