# Supplementary material for: Maternal and offspring intelligence in relation to BMI across childhood and adolescence
Source: Int J Obes (Lond). 2018 Jan 30;42(9):1610–20. doi: 10.1038/s41366-018-0009-1 (PMC6002784; doi:10.1038/s41366-018-0009-1)
Supplement: Supplementary file 8 — Table S7 [file 41366_2018_9_MOESM8_ESM.docx]

Table S7

| Multinomial logistic regression analyses of the relation between an SD increase in IQ and boys’ BMI category across childhood and adolescence adjusting for potential confounding and/or mediating variables. | | | | | | | | | | | | | | |  |  |
| --- | --- | --- | --- | --- | --- | --- | --- | --- | --- | --- | --- | --- | --- | --- | --- | --- |
|  |  | Middle Childhood | | | Late Childhood | | | | Early Adolescence | | | Middle Adolescence | | |  |  |
|  |  |  | Baseline model | Fully adjusted model |  | Baseline model | Fully adjusted model | |  | Baseline model | Fully adjusted model |  | Baseline model | Fully adjusted model |  |  |
|  |  | N | OR (95% CI), P value | OR (95% CI), P value | N | OR (95% CI), P value | OR (95% CI), P value | | N | OR (95% CI), P value | OR (95% CI), P value | N | OR (95% CI), P value | OR (95% CI), P value |  |  |
| **Non-Black & Non-Hispanic** |  |  |  |  |  |  | |  |  |  |  |  |  |  |  |  |
| Boys’ IQ | Under weight | 252 | 1.13 (0.98 to 1.31), 0.096 | 1.02 (0.87 to 1.20), 0.808 | 147 | 0.98 (0.81 to 1.19), 0.854 | | 0.98 (0.79 to 1.22), 0.866 | 85 | 0.92 (0.72 to 1.17), 0.504 | 0.95 (0.72 to 1.26), 0.720 | 37 | 1.00 (0.70 to 1.43), 0.997 | 0.95 (0.63 to 1.45), 0.818 |  |  |
|  | Normal | 1014 | -- | -- | 906 | -- | | -- | 851 | -- | -- | 784 | -- | -- |  |  |
|  | Over weight | 170 | 1.11 (0.93 to 1.30), 0.271 | 1.07 (0.89 to 1.30), 0.459 | 262 | 1.03 (0.89 to 1.20), 0.713 | | 1.07 (0.91 to 1.27), 0.446 | 251 | 1.08 (0.93 to 1.27), 0.289 | 1.16 (0.97 to 1.39), 0.096 | 234 | 0.93 (0.79 to 1.09), 0.366 | 1.00 (0.84 to 1.21), 0.967 |  |  |
|  | Obese | 97 | 1.04 (0.84 to 1.30), 0.714 | 1.04 (0.82 to 1.32), 0.741 | 120 | 0.86 (0.70 to 1.05), 0.152 | | 1.02 (0.81 to 1.30), 0.843 | 138 | 1.01 (0.83 to 1.23), 0.902 | 1.25 (0.99 to 1.57), 0.059 | 135 | 1.02 (0.83 to 1.25), 0.883 | 1.22 (0.96 to 1. 45), 0.100 |  |  |
|  |  |  |  |  |  |  | |  |  |  |  |  |  |  |  |  |
| Mothers’ IQ | Under weight | 252 | **1.22 (1.04 to 1.42), 0.014** | 1.04 (0.84 to 1.27), 0.723 | 147 | 0.92 (0.76 to 1.13), 0.442 | | 0.95 (0.73 to 1.25), 0.701 | 85 | 0.85 (0.66 to 1.11), 0.236 | 0.84 (0.59 to 1.19), 0.325 | 37 | 1.00 (0.68 to 1.48), 0.985 | 0.89 (0.53 to 1.49), 0.648 |  |  |
|  | Normal | 1014 | -- | -- | 906 | -- | | -- | 851 | -- | -- | 784 | -- | -- |  |  |
|  | Over weight | 170 | 1.04 (0.86 to 1.24), 0.680 | 0.86 (0.67 to 1.09), 0.216 | 262 | 0.98 (0.83 to 1.14), 0.773 | | 0.87 (0.70 to 1.07), 0.187 | 251 | 0.95 (0.81 to 1.13), 0.591 | 0.85 (0.68 to 1.05), 0.139 | 234 | 0.91 (0.77 to 1.08), 0.309 | 0.87 (0.69 to 1.08), 0.220 |  |  |
|  | Obese | 97 | 0.95 (0.75 to 1.20), 0.657 | 0.89 (0.64 to 1.22), 0.460 | 120 | **0.79 (0.64 to 0.98), 0.035** | | 0.89 (0.66 to 1.19), 0.420 | 138 | 0.81 (0.66 to 1.00), 0.055 | 0.88 (0.67 to 1.16), 0.363 | 135 | 0.93 (0.75 to 1.15), 0.519 | 1.04 (0.78 to 1.38), 0.806 |  |  |
|  |  |  |  |  |  |  | |  |  |  |  |  |  |  |  |  |
| **Black** |  |  |  |  |  |  | |  |  |  |  |  |  |  |  |  |
| Boys’ IQ | Under weight | 115 | 0.87 (0.70 to 1.08), 0.203 | 0.87 (0.71 to 1.11), 0.267 | 78 | 1.13 (0.88 to 1.45), 0.348 | | 1.20 (0.90 to 1.60), 0.206 | 50 | 1.01 (0.73 to 1.39), 0.946 | 1.03 (0.72 to 1.49), 0.852 | 18 | 1.19 (0.72 to 1.97), 0.500 | 0.99 (0.55 to 1.75), 0.966 |  |  |
|  | Normal | 507 | -- | -- | 521 | -- | | -- | 505 | -- | -- | 507 | -- | -- |  |  |
|  | Over weight | 100 | 1.10 (0.88 to 1.39), 0.406 | 1.09 (0.84 to 1.40), 0.505 | 150 | **1.38 (1.13 to 1.68), 0.001** | | **1.40 (1.12 to 1.75), 0.003** | 180 | 1.07 (0.90 to 1.30), 0.433 | 1.00 (0.81 to 1.23), 0.999 | 182 | 0.98 (0.82 to 1.17), 0.819 | 0.95 (0.77 to 1.16), 0.613 |  |  |
|  | Obese | 71 | 0.98 (0.75 to 1.28), 0.862 | 0.97 (0.71 to 1.31), 0.827 | 91 | 1.16 (0.91 to 1.46), 0.226 | | 1.19 (0.90 to 1.57), 0.224 | 111 | 1.07 (0.86 to 1.35), 0.532 | 1.03 (0.79 to 1.34), 0.815 | 112 | 1.14 (0.91 to 1.42), 0.255 | 1.17 (0.91 to 1.52), 0.217 |  |  |
|  |  |  |  |  |  |  | |  |  |  |  |  |  |  |  |  |
| Mothers’ IQ | Under weight | 115 | 0.87 (0.66 to 1.14), 0.308 | 0.90 (0.64 to 1.27), 0.548 | 78 | 0.89 (0.64 to 1.23), 0.492 | | 0.90 (0.59 to 1.36), 0.624 | 50 | 0.92 (0.62 to 1.38), 0.688 | 0.90 (0.54 to 1.49), 0.669 | 18 | 1.84 (1.00 to 3.42), 0.051 | 1.97 (0.87 to 4.48), 0.102 |  |  |
|  | Normal | 507 | -- | -- | 521 | -- | | -- | 505 | -- | -- | 507 | -- | -- |  |  |
|  | Over weight | 100 | 1.30 (0.97 to 1.72), 0.082 | 1.34 (0.92 to 1.93), 0.121 | 150 | 1.21 (0.94 to 1.54), 0.140 | | 1.01 (0.73 to 1.39), 0.943 | 180 | **1.32 (1.05 to 1.67), 0.019** | **1.35 (1.01 to 1.80), 0.046** | 182 | 1.19 (0.94 to 1.49), 0.139 | 1.15 (0.85 to 1.55), 0.363 |  |  |
|  | Obese | 71 | 0.98 (0.70 to 1.38), 0.927 | 0.70 (0.45 to 1.11), 0.125 | 91 | 1.27 (0.94 to 1.72), 0.116 | | 1.08 (0.72 to 1.63), 0.707 | 111 | **1.34 (1.02 to 1.77), 0.038** | 1.43 (1.00 to 2.05), 0.053 | 112 | 1.19 (0.90 to 1.58), 0.206 | 1.07 (0.74 to 1.55), 0.727 |  |  |
|  |  |  |  |  |  |  | |  |  |  |  |  |  |  |  |  |
| **Hispanic** |  |  |  |  |  |  | |  |  |  |  |  |  |  |  |  |
| Boys’ IQ | Under weight | 93 | 1.13 (0.88 to 1.44), 0.339 | 1.05 (0.80 to 1.38), 0.726 | 50 | 0.76 (0.56 to 1.03), 0.079 | | 0.75 (0.53 to 1.05), 0.092 | 37 | 0.93 (0.64 to 1.34), 0.693 | 0.89 (0.58 to 1.34), 0.565 | 27 | 0.89 (0.58 to 1.36), 0.590 | 0.80 (0.50 to 1.28), 0.355 |  |  |
|  | Normal | 353 | -- | -- | 314 | -- | | -- | 302 | -- | -- | 303 | -- | -- |  |  |
|  | Over weight | 73 | **1.35 (1.03 to 1.78), 0.030** | 1.31 (0.96 to 1.77), 0.090 | 124 | 1.22 (0.97 to 1.52), 0.082 | | 1.26 (0.98 to 1.60), 0.071 | 136 | 1.06 (0.86 to 1.32), 0.560 | 1.13 (0.89 to 1.45), 0.325 | 121 | 0.88 (0.70 to 1.09), 0.251 | 0.89 (0.69 to 1.17), 0.429 |  |  |
|  | Obese | 57 | 0.96 (0.71 to 1.29), 0.795 | 1.02 (0.76 to 1.43), 0.902 | 64 | 1.06 (0.80 to 1.42), 0.656 | | 1.08 (0.79 to 1.48), 0.625 | 71 | 1.05 (0.80 to 1.39), 0.699 | **1.39 (1.00 to 1.93), 0.050** | 78 | 0.93 (0.71 to 1.22), 0.594 | 1.05 (0.77 to 1.44), 0.735 |  |  |
|  |  |  |  |  |  |  | |  |  |  |  |  |  |  |  |  |
| Mothers’ IQ | Under weight | 93 | 1.09 (0.82 to 1.46), 0.549 | 0.88 (0.60 to 1.30), 0.521 | 50 | 0.90 (0.62 to 1.31), 0.587 | | 1.16 (0.70 to 1.93), 0.569 | 37 | 0.87 (0.58 to 1.31), 0.514 | 0.85 (0.49 to 1.49), 0.578 | 27 | 1.16 (0.71 to 1.90), 0.553 | 1.46 (0.75 to 2.83), 0.270 |  |  |
|  | Normal | 353 | -- | -- | 314 | -- | | -- | 302 | -- | -- | 303 | -- | -- |  |  |
|  | Over weight | 73 | 1.20 (0.88 to 1.64), 0.254 | 1.08 (0.71 to 1.65), 0.712 | 124 | 1.09 (0.85 to 1.42), 0.474 | | 1.07 (0.76 to 1.51), 0.709 | 136 | 0.95 (0.75 to 1.22), 0.710 | 0.90 (0.65 to 1.27), 0565 | 121 | 0.98 (0.76 to 1.28), 0.907 | 0.95 (0.67 to 1.35), 0.794 |  |  |
|  | Obese | 57 | **0.66 (0.45 to 0.94), 0.023** | **0.55 (0.34 to 0.89), 0.014** | 64 | 0.97 (0.69 to 1.36), 0.855 | | 1.02 (0.64 to 1.60), 0.948 | 71 | 0.75 (0.55 to 1.02), 0.071 | 0.76 (0.49 to 1.17), 0.210 | 78 | 0.94 (0.69 to 1.28), 0.700 | 0.97 (0.64 to 1.48), 0.900 |  |  |
| *Note*. ^a^ PIAT was the measure of boys’ intelligence | | | | | | | | | | | | | | | | |
| ^b^ AFQT was the measure of mothers’ intelligence.  ^c^ values that are in **bold** are statistically significant.  Baseline Model: PIAT or AFQT & child age  Fully adjusted Model: PIAT, AFQT, child age, mothers' pre-pregnancy BMI, family SES (net family income, year income was recorded, & maternal education). | | | | | | | | | | | | | | | | |
